# The Expression of the Chemokine CXCL14 Correlates with Several Aggressive Aspects of Glioblastoma and Promotes Key Properties of Glioblastoma Cells

**DOI:** 10.3390/ijms20102496

**Published:** 2019-05-21

**Authors:** Barbara Fazi, Carla Proserpio, Silvia Galardi, Francesca Annesi, Mattia Cola, Annunziato Mangiola, Alessandro Michienzi, Silvia Anna Ciafrè

**Affiliations:** 1 Department of Biomedicine and Prevention, University of Rome Tor Vergata, 00133 Rome, Italy; fazi@uniroma2.it (B.F.); carla.proserpio@libero.it (C.P.); silvia.galardi@uniroma2.it (S.G.); francesca.annesi@hotmail.it (F.A.); mattia.cola93@gmail.com (M.C.); alessandro.michienzi@uniroma2.it (A.M.); 2 Department of Neurosurgery, Università degli Studi “G. D’Annunzio”, 65122 Pescara, Italy; annunziato.mangiola@unich.it

**Keywords:** glioblastoma, CXCL14, tumor microenvironment, chemokine

## Abstract

Glioblastoma (GBM) is a primary brain tumor whose prognosis is inevitably dismal, leading patients to death in about 15 months from diagnosis. Tumor cells in the mass of the neoplasm are in continuous exchange with cells of the stromal microenvironment, through the production of soluble molecules, among which chemokines play prominent roles. CXCL14 is a chemokine with a pro-tumor role in breast and prostate carcinoma, where it is secreted by cancer associated fibroblasts, and contributes to tumor growth and invasion. We previously observed that CXCL14 expression is higher in GBM tissues than in healthy white matter. Here, we study the effects of exogenously supplemented CXCL14 on key tumorigenic properties of human GBM cell lines. We show that CXCL14 enhances the migration ability and the proliferation of U87MG and LN229 GBM cell lines. None of these effects was affected by the use of AMD3100, an inhibitor of CXCR4 receptor, suggesting that the observed CXCL14 effects are not mediated by this receptor. We also provide evidence that CXCL14 enhances the sphere-forming ability of glioblastoma stem cells, considered the initiating cells, and is responsible for tumor onset, growth and recurrence. In support of our in vitro results, we present data from several GBM expression datasets, demonstrating that CXCL14 expression is inversely correlated with overall survival, that it is enriched at the leading edge of the tumors and in infiltrating tumor areas, and it characterizes mesenchymal and NON G-CIMP tumors, known to have a particularly bad prognosis. Overall, our results point to CXCL14 as a protumorigenic chemokine in GBM.

## 1. Introduction

Glioblastoma is the most common and deadliest type of brain tumor, that, despite multimodal and aggressive therapy, leads to death within 15 months from diagnosis [1,2]. These tumors, highly infiltrative in the brain parenchyma, are composed not only of purely cancer cells, but also by a variety of stromal cells, among which reactive astrocytes and microglia/macrophages play prominent roles in sustaining tumor growth and progression [3,4,5]. A continuous crosstalk, represented by several types of soluble molecules, connects tumor cells and the surrounding stromal cells in the tumor microenvironment. Chemokines, a family of secreted proteins with established roles in the stimulation of cell migration and growth, are important mediators of tumor-stroma connections in general in solid tumors, and for some of them, a specific function in glioblastoma was demonstrated [6,7]. CXCL14 is a well conserved chemokine with chemoattractive activity for activated macrophages, immature dendritic cells and natural killer cells [8]. Although some papers reported the possible binding of CXCL14 to specific GPCR receptors [8,9], the question is still open about which receptor(s) bind(s) CXCL14 in different contexts. For this reason, this chemokine is still defined as an “orphan” one.

Although a number of studies have demonstrated a tumor-suppressive role for CXCL14 [10,11,12,13], others showed a pro-tumor one [14]. In particular, in prostate and breast cancer, CXCL14 is produced by cancer associated fibroblasts (CAFs) in the tumor microenvironment and contributes to tumor growth and invasion [15,16]. Thus, it appears that CXCL14 action in cancer is dependent on the tumor type. A common feature across all cancers where CXCL14 exerts a tumor-supporting function is its overexpression in the tumor compared to the respective healthy tissue. In glioblastoma, we previously showed that CXCL14 mRNA expression is higher than in healthy white matter [17]. Notably, CXCL14 expression follows a declining pattern, from the frankly tumoral regions, through the peritumoral areas of the same patients, to the healthy tissue, where it is almost undetectable. In patient tissues, we confirmed CXCL14 protein expression by IHC, and provided an indication that it is expressed in the cytosol and extracellular region of reactive astrocytes, strongly infiltrating tumor samples [17]. 

The objective of our present work is to deepen our comprehension of CXCL14 expression and function in glioblastoma cells. To this aim, we performed a series of in vitro experiments to assess if an exogenous source of CXCL14 can affect key properties of glioblastoma cell lines and stem cells. We also analyzed several human glioblastoma datasets for CXCL14 expression, and studied if and how it correlates with some of the main clinical aspects of these tumors.

## 2. Results

**Figure 1:**
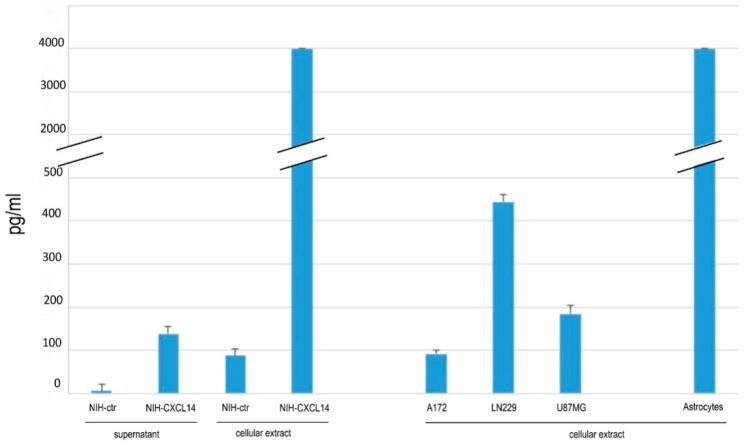
CXCL14 protein expression and secretion. Measurement of CXCL14 protein levels by ELISA in cell protein extracts of cultured human astrocytes, three human glioblastoma cell lines (A172, LN229, U87MG), and in protein extracts and conditioned media of NIH-ctr and NIH-CXCL14 fibroblasts. Results are shown as the mean ± S.D. and represent the average of three biological replicates per sample.

In order to set up our in vitro model, we started by measuring CXCL14 expression in a number of human glioblastoma cell lines, and compared them with cultured human astrocytes. In the cell extracts of three human glioblastoma cell lines, namely A172, LN229 and U87MG, we could detect, by ELISA, CXCL14 levels in the range of 90–400 pg/mL. In support of our previous findings about the enriched expression of CXCL14 in astrocytes in the bulk of the tumor, we found that the cell extracts of cultured human astrocytes contain CXCL14 at a concentration which is at least one order of magnitude higher than that measured in glioblastoma cells (Figure 1).

Our working hypothesis is that CXCL14 in GBM samples is produced mainly by “stromal” cells, such as reactive astrocytes, thus affecting the tumoral properties of GBM cells. In order to establish a source of secreted CXCL14 to be used in in vitro experiments with glioblastoma cells, we took advantage of a cell line of NIH-3T3 fibroblasts stably expressing human CXCL14, NIH-CXCL14 [15]. As expected, these cells not only produced very large human CXCL14 levels, comparable to those endogenously found in human astrocytes, but they also secreted CXCL14 (138.5 pg/mL) in their supernatant (Figure 1). 

**Figure 2:**
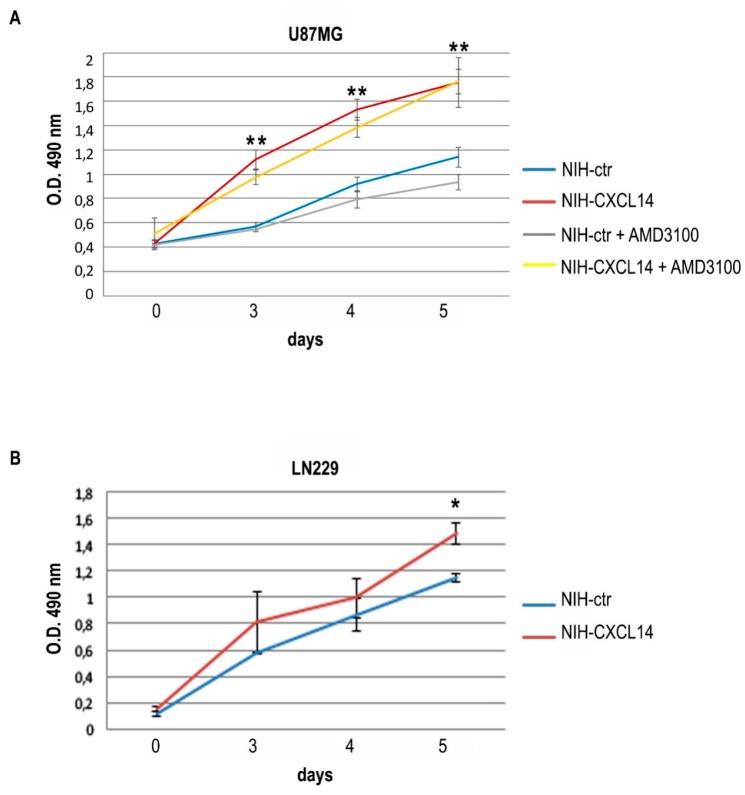
Exogenously supplemented CXCL14 positively affects the proliferation of glioblastoma cells. Proliferation (MTS assay) of U87MG (**A**) or LN229 (**B**) cells grown in the presence of NIH-ctr or NIH-CXCL14 conditioned media. In panel (**A**), the results of the supplementation of 10 μM AMD3100 to U87MG cells incubated with either NIH-ctr or NIH-CXCL14 conditioned media are also shown. Results are shown as the mean ± S.D. and represent the average of three experiments performed independently. Data were analyzed by a two-tailed unpaired Student’s *t*-test. * *p* < 0.05; ** *p* < 0.01.

We chose to employ NIH-CXCL14 conditioned medium as a source of exogenous CXCL14 to study the effects on the proliferation of two human glioblastoma cell lines, LN229 and U87MG, both expressing CXCL14, though at low levels (Figure 1), but unable to produce detectable levels of the secreted chemokine in their supernatants (not shown). The incubation with NIH-CXCL14 conditioned medium sensibly and reproducibly enhanced U87MG cell growth, with an effect which increased over time (Figure 2A). However, NIH-CXCL14 CM barely affected cell growth of LN229 cells, only at late time points, as seen in Figure 2B. 

CXCL14 is considered an “orphan” chemokine, as its receptor has never been unequivocally defined, even if some papers showed that it can bind to CXCR4 [18], formally the receptor of CXCL12. This receptor is expressed on glioblastoma cells and is required for tumor growth, and its stimulation is involved in VEGF production by glioblastoma cells, and in the interaction with endothelial cells in the tumor [19,20,21]. With the aim of understanding if CXCL14 functional effects we observed on glioblastoma cell lines may be mediated by CXCR4, we employed the specific CXCR4 inhibitor AMD3100 [22] in proliferation assays of U87MG cells incubated with NIH-CXCL14 conditioned medium. However, in the presence of AMD3100, the increase in cell proliferation due to NIH-CXCL14 supernatant was maintained (Figure 2A), suggesting that CXCL14 effect on proliferation is not mediated by CXCR4. In addition, we did not observe any variation in CXCR4 expression levels in U87MG cells grown in NIH-CXCL14 conditioned medium, compared to cells grown in NIH-ctr conditioned medium (Supplementary Figure S1), indicating that CXCL14 exogenous supplementation does not affect CXCR4 basal expression.

**Figure 3:**
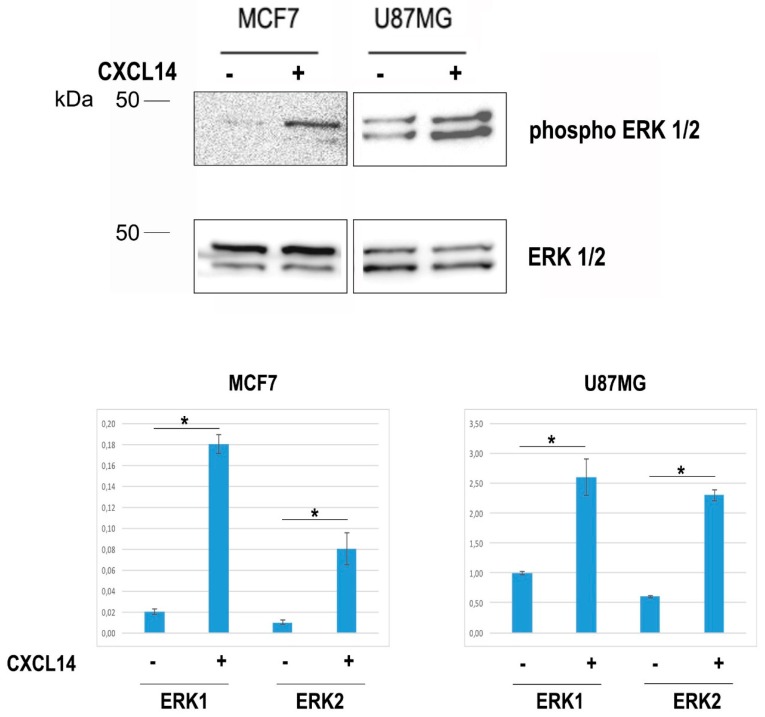
The treatment with CXCL14 induces the phosphorylation of ERK1 and ERK2 in U87MG cells. Representative Western blot showing total (ERK1/2) (**lower panel**) and phosphorylated (phospho-ERK1/2) ERK1/2 (**upper panel**) proteins in total protein extracts of MCF7 or U87MG cells treated or not with recombinant human CXCL14 (400 ng/mL). The graphs show the densitometric quantification of the detected bands, and represent the average (+/− st. dev.) of three independent experiments. The panel relative to MCF7 cells, assayed as positive controls of CXCL14 action on ERK phosphorylation, was produced after a longer exposure, in order to reveal the faint bands present in untreated cells. * *p* < 0.05.

CXCL14 has a demonstrated role as a pro-tumoral chemokine produced in the tumor microenvironment of breast carcinoma by cancer associated fibroblasts (CAFs) [15,16]. In that context, CXCL14 was shown to play its function by stimulating ERK1/2 phosphorylation. In line with this, when we treated U87MG cells with recombinant CXCL14, we detected an increase in ERK1/2 phosphorylated forms (Figure 3).

**Figure 4:**
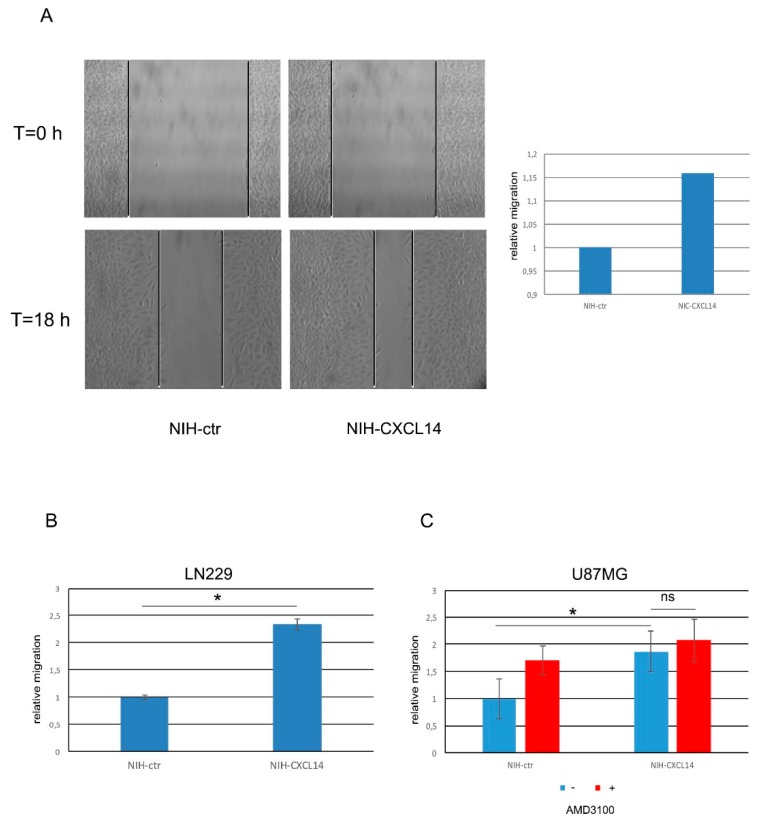
Exogenously supplemented CXCL14 increases the migratory ability of LN229 and U87MG glioblastoma cells. (**A**) Representative picture (left) and relative graphical visualization (right) of a scratch test assay measuring the migration ability of LN229 cells previously incubated with either NIH-ctr or NIH-CXCL14 conditioned medium. Pictures were taken at time 0, when the scratch was performed, and after 18 h from scratching. In the graph, the distance migrated by cells in control conditions is set as = 1. (**B**) Migration transwell assays performed with LN229 cells after incubation with conditioned media of either negative control NIH-ctr or CXCL14 secreting NIH-CXCL14 cells. (**C**) Migration transwell assays performed with U87MG cells after incubation with conditioned media of either negative control NIH-ctr or CXCL14 secreting NIH-CXCL14 cells, with or without the supplementation of 10 μM AMD3100. The graph shows the number of migrating cells as compared to negative control, set as = 1. Results are presented as mean ± S.D. with significant differences from controls (*) shown (*p* < 0.05). Two-tailed unpaired *t*-tests were used to determine significance between groups. The experiments were performed three times (biological replicates).

As the migratory ability of glioblastoma cells is tightly connected with their lethal features, we also assayed if NIH-CXCL14 conditioned medium could modify the migration propensity of GBM cells. Scratch tests performed on LN229 cells demonstrated that NIH-CXCL14 supernatant significantly increased the number of migrated cells compared to those incubated with the conditioned medium of NIH-ctr negative control cells (Figure 4A). Further assays performed by using Boyden chambers confirmed and refined these results in LN229 cells (Figure 4B), and in U87MG cells too (Figure 4C). In both cell types, CXCL14 supplementation by incubating the cells with NIH-CXCL14 conditioned medium increased the number of migrated cells of about twofold. However, as previously noted in proliferation assays, the inhibition of CXCR4 receptor by AMD3100 did not affect CXCL14 pro-migratory function (Figure 4C).

**Figure 5:**
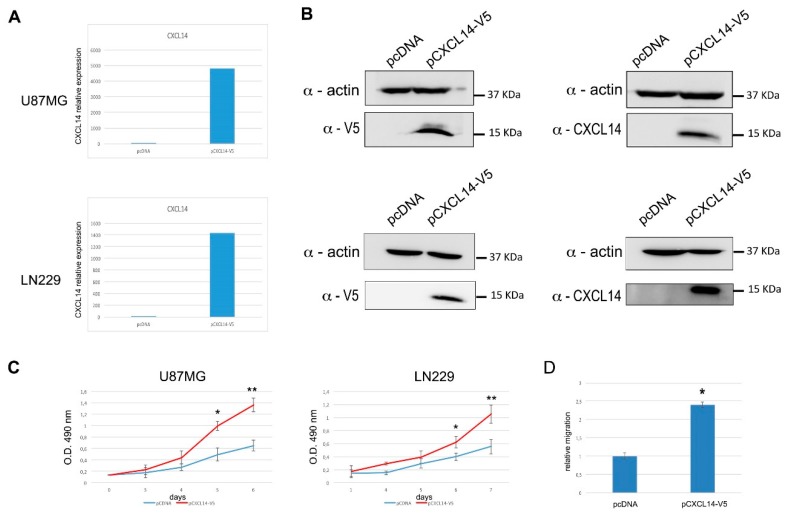
Glioblastoma cells stably overexpressing CXCL14 show enhanced proliferation and migration. (**A**) qRT-PCR analysis of CXCL14 mRNA expression in U87MG (upper panel) or LN229 (lower panel) cells transfected with either the empty vector pcDNA3.1/V5-HisC (pcDNA), or the CXCL14-expressing vector pCXCL14-V5. In both panels, data are expressed as compared to empty vector-transfected cells, where CXCL14 expression was set as = 1. (**B**) Western blot analysis showing CXCL14 protein expression in U87MG (upper panel) or LN229 (lower panel) cells transfected with either the empty vector pcDNA3.1/V5-His C, or the CXCL14-expressing vector pCXCL14-V5. In the immunoblots shown on the left, ectopic CXCL14 was revealed by using an anti-V5 antibody, while on the right a CXCL14-specific antibody was used. β-actin detection was used as a loading control. (**C**) Proliferation (MTS assay) of U87MG (left) or LN229 (right) cells stably transfected with either the empty vector pcDNA3.1/V5-His C, or the CXCL14-expressing vector pCXCL14-V5. Results are shown as the mean ± S.D. and represent the average of two experiments performed in triplicate. Data were analyzed by a two-tailed unpaired Student’s *t*-test. * *p* < 0.05 ** *p* < 0.01. (**D**) Boyden chamber migration assay performed with U87MG cells stably transfected with either the empty vector pcDNA3.1/V5-HisC (pcDNA), or the CXCL14-expressing vector pCXCL14-V5. The graph depicts the relative migration of CXCL14-expressing cells compared to empty vector transfected ones, whose migration ability was set as = 1. Results are presented as mean ± S.D. with significant differences from controls (*) shown (*p* < 0.05).

With the aim of strengthening these results, obtained in transient conditions, we also produced U87MG and LN229 cells stably overexpressing human CXCL14 (Figure 5A,B). Both stable cell lines clearly showed an increased proliferation compared to cells transduced with a negative control vector (Figure 5C). Moreover, the stable U87MG cells overexpressing CXCL14 showed a significantly increased migration ability (Figure 5D) in agreement with what we have shown for LN229 cells.

**Figure 6:**
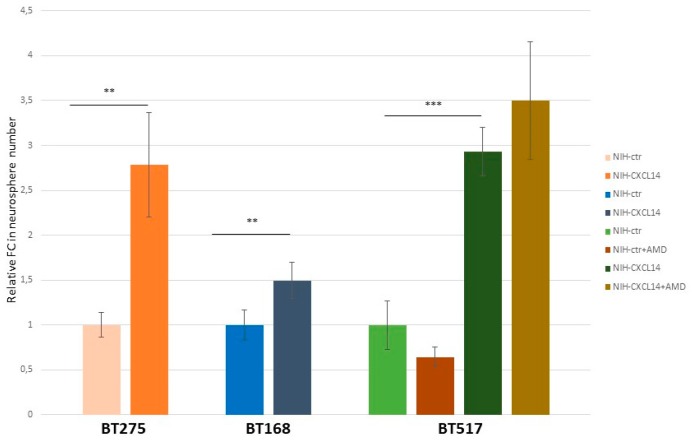
Exogenously supplemented CXCL14 enhances the self-renewal ability of glioblastoma stem cells. Glioblastoma stem cell self-renewal assay. The histogram shows the fold change (FC) in numbers of neurospheres formed by three different glioblastoma stem cell lines, BT275, BT168, and BT517. The results were obtained by counting the number of neurospheres in four random fields per sample, and comparing cells grown in the presence of NIH-ctr conditioned medium to cells grown in the presence of NIH-CXCL14 conditioned medium. For BT517 cells, the assay was performed in either the presence or the absence of AMD3100 (*n* = 3; mean ± S.D.). ** *p*-value < 0.01; *** *p*-value < 0.001.

The origin of glioblastoma, though controversial, is believed to reside in “stem-like” cells, also dubbed as tumor initiating cells; characterized by the ability of self-renewal in vitro and in vivo, and considered to be the main source of glioblastoma resistance to therapy and consequently of tumor relapse and patient death [23,24]. These cells, isolated from fresh tumor samples, can be propagated in vitro and grown as “spheres” in defined culture conditions. In order to extend the frame of our functional observations about CXCL14 possible roles in glioblastoma, we measured the ability of three distinct glioblastoma stem cell lines to form spheres in the presence of NIH-CXCL14 conditioned medium. Figure 6 shows that the incubation with CXCL14 containing medium increased this ability in all cell lines. However, the average size (diameter) of the spheres produced in the two conditions was not significantly different (not shown), suggesting that exogenously supplemented CXCL14 mostly works on the self-renewal ability of GSCs, rather than on their proliferation (reflected by the size of the spheres). In the case of BT517, which showed the highest Fold Change difference in the number of neurospheres produced, we also assayed if AMD3100 could affect the result. Again, as for proliferation and migration of U87MG cells, AMD3100 supplementation did not modify the effect of CXCL14 containing medium on glioblastoma stem cell spherogenic ability.

**Figure 7:**
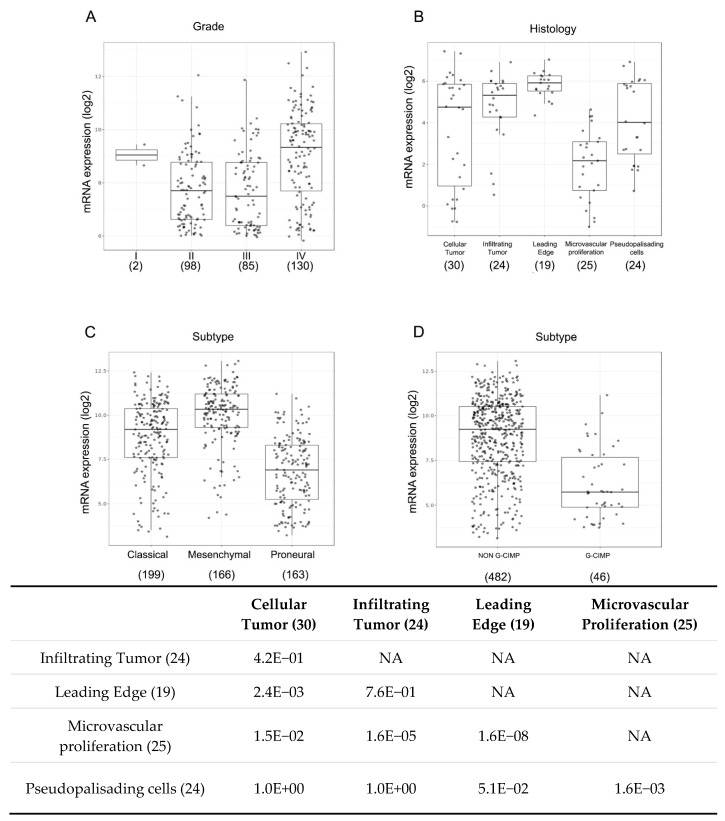
CXCL14 expression correlates with aggressive features in glioblastoma. (**A**) Box-plots of CXCL14 expression in the Rembrandt dataset including 315 gliomas of different grades, as analyzed by GlioVis (http://gliovis.bioinfo.cnio.es/) [25]. GBM IV versus GBM II *p* = 1.2E−08; GBM IV versus III *p* = 7.8E−09. Pairwise comparisons between group levels with corrections for multiple testing (*p*-values with Bonferroni correction). (**B**) Box-plots representing the GlioVis (http://gliovis.bioinfo.cnio.es/) [25] analysis of CXCL14 expression in the IVY GAP dataset, containing RNA-seq results of a total of 122 RNA samples of five anatomic structures generated from 10 glioblastomas (as in the table below, which shows the pairwise comparisons between group levels with corrections for multiple testing (*p*-values with Bonferroni correction)). (**C**,**D**) Box-plot graphs representing the GlioVis (http://gliovis.bioinfo.cnio.es/) [25] analysis of CXCL14 expression in the TCGA dataset, including glioblastoma samples from 528 patients, whose tumors were classified as “classical” CL, mesenchymal” MES or “proneural” PN (in C) (MES versus CL *p* = 3.8 × 10^−9^; MES *vs* PN *p* = 2.7 × 10^−42^; CL *vs* PN *p* = 1.5 × 10^−19^) or as NON G-CIMP and G-CIMP (in D) (NON G-CIMP *vs* G-CIMP *p* = 2.8 × 10^−14^). Pairwise comparisons between group levels with corrections for multiple testing (*p*-values with Bonferroni correction). In all graphs, for each group of samples, the number in brackets represents the number of samples analyzed.

To complement our experimental results with a view of clinical data depicting CXCL14 expression in glioblastoma samples, we analyzed several datasets of glioblastoma patients by mining GlioVis, a web application for data visualization and analysis of brain tumors expression datasets (http://gliovis.bioinfo.cnio.es/) [25]. In the Rembrandt dataset, including 315 gliomas of different grades, the highest CXCL14 expression is found in grade IV tumors, the most aggressive and lethal ones (Figure 7A). In addition, mining of the IVY GAP dataset, containing RNA-seq results of a total of 122 RNA samples of 5 anatomic structures generated from 10 tumors, revealed that CXCL14 expression is stronger at the leading edge of the tumors and also in infiltrating tumor areas, compared to other parts of the tumors (Figure 7B). We also found strong evidence of a subtype-specific enrichment of CXCL14 expression in glioblastoma. In fact, as shown in Figure 7C, CXCL14 RNA is clearly overexpressed in mesenchymal tumors, compared to classical (*p* = 3.8 × 10^−9^) and even more to proneural ones (*p* = 2.7 × 10^−42^). In the same dataset (TCGA, including 528 patients), CXCL14 expression neatly characterizes NON G-CIMP samples (NON G-CIMP vs G-CIMP, *p* = 2.8 × 10^−14^) (Figure 7D).

**Figure 8:**
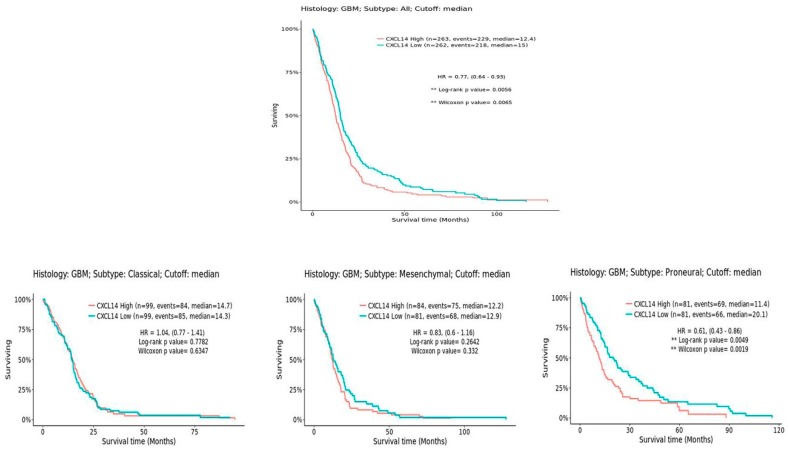
CXCL14 expression inversely correlates with overall survival in glioblastoma. On the left, a comprehensive graph shows the overall survival of all glioblastoma patients in the TCGA dataset, as analyzed by GlioVis (http://gliovis.bioinfo.cnio.es/) [25]; on the right, three distinct graphs were generated by separately analyzing GBM patients based on the subtypes of their tumors.

Notably, patients carrying NON G-CIMP tumors are known to have a worse prognosis than G-CIMP ones [26]. Interestingly, CXCL14 expression shows an inverse correlation with overall survival in this set of patients (Figure 8). However, when patients are divided by the subtype of tumors, it is clear that the statistically different survival is observed specifically only in the proneural subtype (Figure 8), where the overall levels of CXCL14 are the lowest compared to all other subtypes. This suggests that CXCL14 expression may discriminate, among proneural glioblastomas, those with the worst prognosis.

Altogether, these observations point to CXCL14 expression correlating with the most aggressive types of glioblastomas and to the most aggressive regions in the tumor mass, those that drive the growth and dissemination of the tumor in the affected brain.

## 3. Discussion

The role of CXCL14 in cancer is controversial, as it was shown to play either tumor suppressive or tumor supportive roles, depending on the specific tumor type [13,27,28,29,30]. In glioblastoma, however, others and us showed that CXCL14 mRNA expression is enriched in tumor samples [17,31], where this chemokine is likely produced and secreted in the tumor microenvironment by reactive astrocytes [17] or other types of stromal cells [32]. To this regard, it is interesting to notice that, in the samples we tested in the present work, the most abundant expression of CXCL14 was found in cultured astrocytes. Notwithstanding the differences obviously existing between reactive astrocytes in vivo and cultured astrocytes, this may anyway suggest that astrocytes own the intrinsic ability to efficiently produce CXCL14. These findings indicate that CXCL14 may contribute to the tumor supportive function of the microenvironment, ultimately strengthening the aggressive features of glioblastoma cells. Here, we show that indeed CXCL14, both exogenously supplemented and endogenously overexpressed, enhances the proliferation and the migratory ability of two different cell lines of human glioblastoma. In these cells, the exogenous supplementation of CXCL14 induces ERK1/2 phosphorylation, as it does in breast carcinoma, where CXCL14 is secreted in the microenvironment by cancer associated fibroblasts [15]. This result too converges with those collected in other solid tumors, where CXCL14 plays a tumor supportive role. Moreover, our observation that CXCL14 enhances the ability of glioblastoma stem cells to form neurospheres is completely new, and indicates that this chemokine may contribute to key functions of the cells at the origin of glioblastoma. However, what remains still obscure is the receptor through which this chemokine signals and produces its downstream effects. In our model, it is unlikely that CXCR4 is involved, as the specific inhibition of this receptor did not modify CXCL14 effects on glioblastoma cells. We also think we can reasonably exclude that GPR85, an orphan G protein-coupled receptor recently found to bind CXCL14 on mammary fibroblasts in the tumor microenvironment of breast carcinoma [9], works as the receptor for CXCL14 in glioblastoma, as our SAGE data in glioblastoma samples and an RNA-seq analysis on GBM stem-like cells we recently performed, did not show any evidence of GPR85 expression in tumor samples [17] and data not shown. During the preparation of our manuscript, a paper was published, which describes the role of fibroblast-derived CXCL14 in EMT and metastasis of breast cancer [33], and identifies the GPCR ACKR2 as a mediator for CXCL14 action. In the glioblastoma cells we are studying here, though, we do not deem it likely that CXCL14 functions are mediated by ACKR2, as the expression of its mRNA, that we measured by qRTPCR, was undetectable in both U87MG and LN229 cells (not shown). Moreover, as for GPR85, our SAGE data in glioblastoma samples and an RNA-seq analysis on GBM stem-like cells we recently performed, showed only a barely detectable level of ACKR2 mRNA expression in tumor samples [17] and data not shown. What Sjöberg and co-authors show however, may be still highly relevant to glioblastoma too, as the exogenous source of CXCL14 they use is the same as ours, i.e., murine fibroblasts ectopically overexpressing human CXCL14. In their paper, they identify soluble factors that are induced in the secretome of NIH-CXCL14 fibroblasts compared to negative control ones, and propose that some of the molecules specifically enriched in those cells, among which many pro-angiogenic factors, inducers of EMT and of extracellular matrix remodeling, and several other chemokines, may be the direct effectors of CXCL14 action on tumor cells. Notably, among these molecules, several are known to positively affect the tumorigenic properties of glioblastoma cells. Thus, we cannot exclude that in glioblastoma too, CXCL14 exerts its pro-tumoral role at least in part indirectly, by inducing, in the producer cells of the microenvironment, the secretion of other molecules. In our work however, we also show that CXCL14 produced directly by stably overexpressing glioblastoma cell lines, enhances their proliferation and migration ability. Thus, CXCL14 produced in a completely different context from the fibroblast-derived one, still induces the same results. We think that it is quite unlikely that different cell types (murine fibroblasts versus human glioblastoma cell lines) respond to ectopic CXCL14 overexpression by secreting the same set of factors, even though we cannot exclude that some of them may overlap, and be at least in part responsible for CXCL14 action. In fact, in the absence of commercially available bona fide anti-CXCL14 blocking antibodies, we cannot discriminate between a purely direct and a partly indirect mechanism of action for CXCL14 on glioblastoma cells.

Regarding the clinical implications of our results, our findings about CXCL14 expression in glioma datasets support a view of CXCL14 tagging the most aggressive tumors (IV grade versus other grades), subtypes (mesenchymal and classical versus proneural), regions (leading edge of the tumors and infiltrating tumor areas), and G-CIMP status (NON G-CIMP vs G-CIMP). This last observation in particular may overlap with that, by Zeng and co-authors [31], about a trend of CXCL14 overexpression in IDH wt gliomas *vs* IDH mutant ones: G-CIMP tumors in fact were closely related to IDH mutation, and a better prognosis characterizes patients carrying them [26,34]. Moreover, G-CIMP subtypes are highly enriched among the proneural subtypes, compared with NON G-CIMP tumors [34]. However, a detailed analysis showed that, among IDH mutant gliomas, the extent of global DNA methylation can be used to further distinguish patients: tumors with a low degree of DNA methylation (G-CIMP-low) presented a poorer outcome, while those with higher DNA methylation (G-CIMP-high) had a better overall survival [35]. It may be interesting to verify if, among the proneural tumors, those we have found with higher CXCL14 expression and shorter survival, correspond to the G-CIMP-low subset. In this view, our observation that CXCL14, much less expressed in the proneural subtype of tumors than in the others, shows an inverse correlation with overall survival—specifically in that subtype, may be of particular interest. Moreover, proneural tumors, even if showing a trend toward a slightly longer survival than other subtypes, do not respond to aggressive therapy [36], defined as concurrent chemo- and radiotherapy or more than three subsequent cycles of chemotherapy. It may be worth investigating if CXCL14 expression in this subtype may affect patients’ response to this therapeutic regimen. 

Altogether, our results highlight the involvement of CXCL14 in glioblastoma environment, where it likely affects not only established tumor cells, but also the stem cells. This observation, together with the subtype-specific enrichment of CXCL14, deserves further studies to unravel the significance of CXCL14 expression in the origin of glioblastoma and in the differential response of these tumors to therapy. To this aim, in vivo studies are needed to unequivocally demonstrate to which extent CXCL14 contributes to GBM growth and, consequently, if it could be considered as a therapeutic target. Further important issues that need to be addressed are the identification of its receptor in glioblastoma and the production of blocking molecules for both CXCL14 and its receptor, in order to selectively prevent its function.

## 4. Materials and Methods

### 4.1. Cell Culture, Transfections and Treatments

The human glioblastoma cell lines A172, LN229, and U87MG (ATCC, Rockville, MD, USA), and the murine fibroblast cell lines NIH-CXCL14 and NIH-ctr (a kind gift of Dr. Arne Östman, Karolinska Institutet, Stockholm, Sweden) were cultured in Dulbecco’s modified Eagle’s medium supplemented with 10% fetal bovine serum (Aurogene, Rome, Italy) in a humidified atmosphere containing 5% CO_2_ at 37 °C. Primary human astrocytes (ScienCell #1800-5-SC) were purchased from CliniSciences (Guidonia Montecelio, Italy) and cultured in Astrocyte Medium (ScienCell #1801). Glioblastoma stem-like cells (kindly provided by Dr. Gaetano Finocchiaro, Istituto Besta, Milan, Italy) were previously described [37,38]. They were grown as neurospheres in standard medium containing: DMEM/F-12 (Life Technologies-GIBCO, Monza, Italy), 2 mM glutamine (Sigma, Milan, Italy), B-27 (1:50, Life Technologies-GIBCO, Monza, Italy), human recombinant fibroblast growth factor 2 (bFGF, 20 ng/mL; Peprotech, London, UK) and epidermal growth factor (EGF, 20 ng/mL; Peprotech, London, UK). For self-renewal assays, GSCs were seeded in 24-well plates at 20000 cells/well in the presence of conditioned media from either NIH-ctr or NIH-CXCL14 fibroblasts, collected after 48 h of growth in the absence of serum. The neurosphere number was counted after 7 days in four random fields per sample. Where indicated, 10 μM AMD3100 (Sigma-Aldrich, #A5602) was added to the medium.

Transfections were performed by Lipofectamine 3000 reagent (Invitrogen, Monza, Italy) in Opti-MEM I (Invitrogen, Monza, Italy), by following the manufacturer’s recommendations. The generation of stably transfected U87MG and LN229 cell lines was obtained by growing cells in medium containing the selective agent, G418, 1 mg/mL for three weeks starting at 48 h after transfection. The construct encoding for human CXCL14 was prepared by cloning human CXCL14 cDNA into pcDNA3.1/V5-His C (Invitrogen) digested with BamHI and XhoI. The primers used for cloning CXCL14 cDNA were: 5′-AAAGGATCCATGTCCCTGCTCCCACGC-3′ and 5′-AAACTCGAGCTTCTTCGTAGACCCTGCG-3′.

For treatments with recombinant human CXCL14 (Peprotech), cells were incubated for 7 minutes with 400 ng/mL recombinant CXCL14, and then collected. 

### 4.2. RNA Isolation, Reverse Transcription and Quantitative Real Time Polymerase Chain Reaction (RT-qPCR) 

Total cellular RNA was harvested with TRIzol LS Reagent^®^ (Life Technologies, Monza, Italy), treated with DNase (New England Biolabs, Ipswich, CA, USA) for 40 min at 37 °C, and reverse transcribed with M-MLV Reverse Transcriptase (Invitrogen), according to the manufacturer’s protocols. RT-qPCR was performed on the Applied BiosystemsStepOne plus PCR System with SsoAdvanced™ Universal SYBR^®^ Green Supermix (Biorad, Segrate, Italy) and the following primers: CXCL14 5′CCCAAGCTGCAGAGCACC3′ and 5′TAGACCCTGCGCTTCTCGTT3′; CXCR4 FW 5′-CTTCATCTTTGCCAACGTCAG-3′ and CXCR4 REV 5′- GGACAGGATGACAATACCAGG-3′; β-ACTIN 5′ACCGAGCGCGGCTACAG3′ and 5′CTTAATGTCACGCACGATTTC3′. The relative amount of each substrate was calculated by the 2^−ΔΔCT^ method.

### 4.3. Human CXCL14 ELISA 

The CXCL14 protein level in protein extracts and conditioned media was determined using Human CXCL14 PicoKine^TM^ ELISA Kit (BOSTER, Pleasanton, CA, USA) following the manufacturer’s protocol. The absorbance at 450 nm was evaluated using a BP800 Microplate Absorbance Reader (Biohit Oyj Healthcare, Milan, Italy). The CXCL14 concentration in the samples was interpolated from the standard curve.

### 4.4. Protein Extraction and WB Analysis 

For Western blot analysis, total protein extract was isolated from A172 and LN229 cells by lysis in NP-40 Buffer containing protease and phosphatase inhibitors (complete EDTA-free; Roche Applied Science, Monza, Italy), and protein concentrations were determined by Bradford method. Equivalent amounts of protein extract were separated by electrophoresis on 10% or 12% SDS-PAGE gels and blotted onto nitrocellulose. The membranes were blocked with 5% non-fat dry milk and 0.1% Tween-20 in Phosphate-buffered saline and then incubated with antibodies followed by appropriate horseradish peroxidase-conjugated secondary antibodies (Promega, Milan, Italy). The primary antibodies employed for protein detection were: anti-CXCL14 #MAB866, R&D Systems (1:500); anti-V5 #R960-25, Invitrogen (1:5000); anti-β-actin #A2066, Sigma (1:10,000); anti-ERK1/2 #4695, Cell Signaling (1:1000) and anti-phospho ERK1/2 #9106, Cell Signaling (1:2000).

### 4.5. Migration Assays

To measure the migration of LN229 cells by a “scratch” test, 150 × 10^3^ cells per well were plated in triplicate in a 24-well plate. After 24 h, the medium of each well was replaced with either NIH-CXCL14 or NIH-ctr conditioned medium, and cells were incubated for additional 30 h in a humidified atmosphere containing 5% CO_2_ at 37 °C. After 30 h, a scratch was produced in each well by a pipette tip, and the medium was changed to serum-free DMEM. Pictures were taken at growing times from the scratch, ending at 18 h.

For the transwell migration assays, U87MG cells stably expressing CXCL14 cells were plated in DMEM culture medium without serum on BD BioCoatControlCell Culture Insert Systems (BD Biosciences, Milan, Italy) at 25 × 10^3^ cells/chamber. The chemoattractant (DMEM supplemented with 10% FBS) was added to the bottom wells of the plate. The cells were incubated at 37 °C, 5% CO_2_, for 6 h. After incubation, the non-migrated cells were removed from the upper surface of the membrane by scrubbing with a cotton tipped swab, while the cells migrated adhering to the bottom surface of the membrane were fixed with 100% MetOH and stained with DAPI. The number of migrated cells was evaluated in three different fields of three different wells for each condition. When the transwell assays were performed with non-transfected LN229 (or U87MG) cells, upon incubation with NIH-ctr or NIH-CXCL14 conditioned media, cells were pre-incubated for 24 h with the conditioned media from either NIH-ctr or NIH-CXCL14 fibroblasts. Then, after washing with PBS, the assay was performed as described above for stable cell lines. Where indicated, 10 μM AMD3100 (Sigma-Aldrich, #A5602) was added to the medium.

### 4.6. MTS Cell Proliferation Analysis

U87MG and LN229 cells were trypsinized, harvested and seeded onto 96-well flat-bottomed plates at a density of 3000 cells/well, then incubated at 37 °C for 24 h in DMEM supplemented with 10% FBS. Then, cells were washed with PBS and the medium was replaced with the conditioned media from either NIH-ctr or NIH-CXCL14 fibroblasts. Subsequently, the cells were subjected to CellTiter 96^®^ AQueous One Solution Cell Proliferation Assay (Promega, Milan, Italy), according to the manufacturer’s protocol at increasing times after conditioned media supplementation. The absorbance at 490 nm was evaluated using a BP800 Microplate Absorbance Reader (Headquarters Biohit Oyj Healthcare, Milan, Italy). Where indicated, 10 μM AMD3100 (Sigma-Aldrich, #A5602) was added to the medium.

### 4.7. Statistical Analysis

Paired *t*-test and GraphPad Prism version 5.00 (GraphPad software, San Diego, CA, USA; http://www.graphpad.com) were used. A *p*-value (*p*) < 0.05 was considered statistically significant. Data were obtained from independent experiments (*n* = 3) and expressed as means ± S.D.

## Supplementary Materials

Supplementary materials can be found at https://www.mdpi.com/1422-0067/20/10/2496/s1.
