# Supplementary figures and images for: The Expression of the Chemokine CXCL14 Correlates with Several Aggressive Aspects of Glioblastoma and Promotes Key Properties of Glioblastoma Cells
Source: Int J Mol Sci. 2019 May 21;20(10):2496. doi: 10.3390/ijms20102496 (PMC6566570; doi:10.3390/ijms20102496)

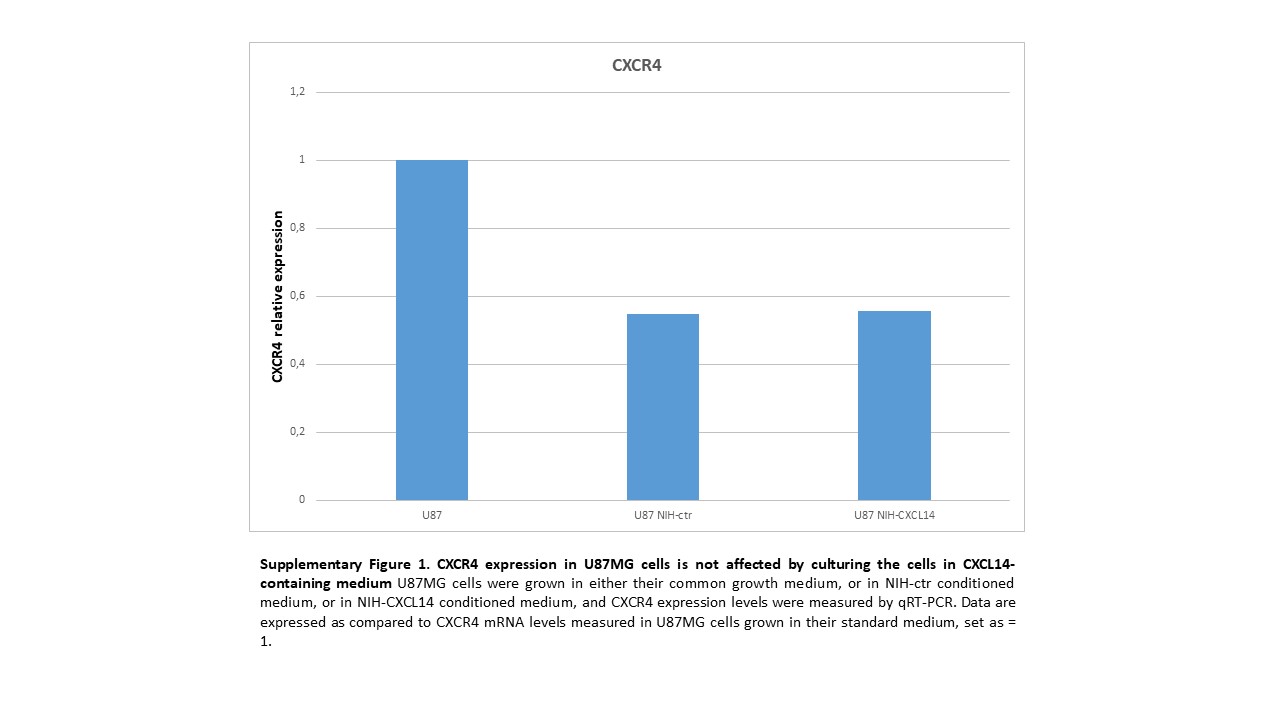

Supplement: Supplementary file 1 [file ijms-20-02496-s001.zip › ijms-501318-supplementary.JPG]
